# Potential Utility of C-reactive Protein for Tuberculosis Risk Stratification Among Patients With Non-Meningitic Symptoms at HIV Diagnosis in Low- and Middle-income Countries

**DOI:** 10.1093/ofid/ofae356

**Published:** 2024-07-03

**Authors:** Kathryn Dupnik, Vanessa R Rivera, Nancy Dorvil, Yanique Duffus, Hanane Akbarnejad, Yipeng Gao, Jingyi Liu, Alexandra Apollon, Emelyne Dumont, Cynthia Riviere, Patrice Severe, Kerlyne Lavoile, Maria Alejandra Duran Mendicuti, Samuel Pierre, Vanessa Rouzier, Kathleen F Walsh, Anthony L Byrne, Patrice Joseph, Pierre-Yves Cremieux, Jean William Pape, Serena P Koenig

**Affiliations:** Center for Global Health, Department of Medicine, Weill Cornell Medicine, New York, New York, USA; Haitian Group for the Study of Kaposi's Sarcoma and Opportunistic Infections (GHESKIO), Port-au-Prince, Haiti; Haitian Group for the Study of Kaposi's Sarcoma and Opportunistic Infections (GHESKIO), Port-au-Prince, Haiti; Center for Global Health, Department of Medicine, Weill Cornell Medicine, New York, New York, USA; The Analysis Group, Boston, Massachusetts, USA; The Analysis Group, Boston, Massachusetts, USA; The Analysis Group, Boston, Massachusetts, USA; Haitian Group for the Study of Kaposi's Sarcoma and Opportunistic Infections (GHESKIO), Port-au-Prince, Haiti; Haitian Group for the Study of Kaposi's Sarcoma and Opportunistic Infections (GHESKIO), Port-au-Prince, Haiti; Haitian Group for the Study of Kaposi's Sarcoma and Opportunistic Infections (GHESKIO), Port-au-Prince, Haiti; Haitian Group for the Study of Kaposi's Sarcoma and Opportunistic Infections (GHESKIO), Port-au-Prince, Haiti; Haitian Group for the Study of Kaposi's Sarcoma and Opportunistic Infections (GHESKIO), Port-au-Prince, Haiti; Brigham and Women's Hospital, Harvard Medical School, Boston, Massachusetts, USA; Haitian Group for the Study of Kaposi's Sarcoma and Opportunistic Infections (GHESKIO), Port-au-Prince, Haiti; Center for Global Health, Department of Medicine, Weill Cornell Medicine, New York, New York, USA; St. Vincent's Hospital and Clinical School, University of New South Wales, Darlinghurst, New South Wales, Australia; Center for Global Health, Department of Medicine, Weill Cornell Medicine, New York, New York, USA; St. Vincent's Hospital and Clinical School, University of New South Wales, Darlinghurst, New South Wales, Australia; Haitian Group for the Study of Kaposi's Sarcoma and Opportunistic Infections (GHESKIO), Port-au-Prince, Haiti; The Analysis Group, Boston, Massachusetts, USA; Center for Global Health, Department of Medicine, Weill Cornell Medicine, New York, New York, USA; St. Vincent's Hospital and Clinical School, University of New South Wales, Darlinghurst, New South Wales, Australia; Brigham and Women's Hospital, Harvard Medical School, Boston, Massachusetts, USA

**Keywords:** C-reactive protein, diagnosis, HIV, rapid treatment initiation, tuberculosis

## Abstract

**Background:**

The World Health Organization recommends initiating same-day antiretroviral therapy (ART) while tuberculosis (TB) testing is under way for patients with non-meningitic symptoms at HIV diagnosis, though safety data are limited. C-reactive protein (CRP) testing may improve TB risk stratification in this population.

**Methods:**

In this baseline analysis of 498 adults (>18 years) with TB symptoms at HIV diagnosis who were enrolled in a trial of rapid ART initiation in Haiti, we describe test characteristics of varying CRP thresholds in the diagnosis of TB. We also assessed predictors of high CRP as a continuous variable using generalized linear models.

**Results:**

Eighty-seven (17.5%) participants were diagnosed with baseline TB. The median CRP was 33.0 mg/L (interquartile range: 5.1, 85.5) in those with TB, and 2.6 mg/L (interquartile range: 0.8, 11.7) in those without TB. As the CRP threshold increased from ≥1 mg/L to ≥10 mg/L, the positive predictive value for TB increased from 22.4% to 35.4% and negative predictive value decreased from 96.9% to 92.3%. With CRP thresholds varying from <1 to <10 mg/L, a range from 25.5% to 64.9% of the cohort would have been eligible for same-day ART and 0.8% to 5.0% would have untreated TB at ART initiation.

**Conclusions:**

CRP concentrations can be used to improve TB risk stratification, facilitating same-day decisions about ART initiation. Depending on the CRP threshold, one-quarter to two-thirds of patients could be eligible for same-day ART, with a reduction of 3- to 20-fold in the proportion with untreated TB, compared with a strategy of same-day ART while awaiting TB test results.

Global guidelines recommend antiretroviral therapy (ART) initiation on the same day of HIV diagnosis for patients who are ready to start treatment [1–4]. These guidelines have been implemented in response to several clinical trials and cohort studies, which have demonstrated improved outcomes and faster time to viral suppression with this approach [5–9]. However, a substantial proportion of patients in high burden settings present with symptoms of tuberculosis (TB) at HIV diagnosis [10, 11]. The World Health Organization (WHO) recommends a 4-symptom screen (W4SS) for cough, fever, night sweats, and/or weight loss before ART initiation, followed by rapid molecular TB testing in symptomatic patients [1]. However, it is logistically challenging to complete a TB evaluation in 1 day because the Xpert MTB/RIF assay (Cepheid, Sunnyvale, CA) has at least a 2-hour turnaround time, and tests are often conducted in centralized laboratories [12]. Furthermore, the specificity of the W4SS is low (42% in a recent meta-analysis); consequently, this approach results in additional visits for test results and delays in ART initiation for patients who do not have active TB. This also increases the number of confirmatory tests that must be conducted by the laboratory, which may delay results for patients who do have TB, contributing to losses in the TB testing cascade.

To avoid delays in ART initiation, the current WHO guidelines include a clinical consideration to initiate same-day ART while investigating for TB in patients with presumptive TB (without symptoms of central nervous system involvement) [1]. However, data on the safety of initiating ART in the presence of undiagnosed TB are limited [13]. Multiple studies of same-day ART initiation have been conducted, but they included only a small number of patients with active TB [6, 8, 10, 12, 13]. Initiating ART before TB treatment may result in increased rates of immune reconstitution inflammatory syndrome, which is associated with substantial morbidity [14, 15].

An alternative approach would be to conduct a rapid point-of-care test for symptomatic patients at HIV diagnosis to further improve TB risk profiling, so that TB testing can be prioritized for high-risk patients, and immediate ART can be initiated in low-risk patients. Rapid point-of-care C-reactive protein (CRP) tests could be particularly well suited for this indication because they are inexpensive ($2 USD/test), generate results in 3 minutes, and can be performed with a fingerstick specimen [16]. Multiple studies have reported that CRP has superior diagnostic characteristics for the detection of TB at the time of HIV diagnosis, compared with the W4SS alone [17–20]. The WHO conditionally recommends the use of CRP with a cutoff of 5 mg/L to screen for active TB in people with HIV, with the caveat that more data are needed [1]. However, CRP testing is not widely available in HIV clinics and is not included in decision-making algorithms about the timing of ART initiation. Therefore, we conducted a study to evaluate the association between CRP levels and active TB among symptomatic patients at HIV diagnosis at the Haitian Group for the Study of Kaposi's Sarcoma and Opportunistic Infections (GHESKIO) in Port-au-Prince, Haiti.

## METHODS

This study included data from a previously published trial, in which persons with non-meningitic TB symptoms at HIV diagnosis were randomized to receive same-day treatment (same-day TB testing with same-day TB treatment if TB diagnosed; same-day ART if TB not diagnosed) versus standard care (initiating TB treatment within 7 days and delaying ART to day 7 if TB not diagnosed) [11]. Patients were eligible for study inclusion if they were infected with HIV-1, ≥18 years of age, nonpregnant, ART-naïve, and reported cough, fever, and/or night sweats of any duration, and/or weight loss that was confirmed by the study physician at presentation to GHESKIO, in Port-au-Prince, Haiti.

At HIV diagnosis, complete blood count (Abbott, Abbott Park, IL), CD4 count (FACSCount, Becton Dickinson, Franklin Lakes, NJ), digital chest radiograph, Xpert Ultra (Cepheid, Sunnyvale, CA), and liquid mycobacterial culture (Mycobacteria Growth Indicator Tube, BACTEC, Becton Dickinson) were conducted for all participants; Xpert Ultra and mycobacterial culture were performed on both spot and early morning specimens. Sera were stored for CRP testing; specimens were processed within 4 hours of collection, biobanked at −80 °C, and shipped to Weill Cornell Medical College for testing (CRP Quantikine ELISA Kit, R&D Systems, Minneapolis, MN). The manufacturer's protocol was followed with the following specifications. Serum samples were thawed and serially diluted 1:100 followed by 1:5 to make a final dilution of 1:500 for each sample. The 1:100 dilution was saved for subsequent tests if samples needed to be repeated. Absorbance was measured at 450 nm with 540 nm correction. A standard curve (minimum, 3.9 mg/L; maximum, 25 mg/L) and 3 positive controls with varying concentration were included in each assay performed. Samples that had a CRP reading above 25 mg/L were retested at a 1:2500 or 1:10 000 dilution for each sample.

CRP testing was conducted retrospectively, so results were not available to guide decision making. All participants in both groups received initial TB test results before ART or TB treatment initiation; participants in the same-day treatment group received same-day TB test results, which is not standard of care at GHESKIO.

This study was approved by the institutional review boards at GHESKIO, Mass General Brigham, and Weill Cornell Medical College. Written informed consent was obtained from all participants.

### Statistical Analysis

Demographic, clinical, and laboratory information were extracted from the GHESKIO electronic medical record. Baseline characteristics were summarized by tuberculosis status using medians and interquartile ranges (IQR) for continuous variables and counts and percentages for categorical variables. Age, body mass index (BMI), hemoglobin, and CD4 count were continuous variables. Sex, income, education, TB symptoms, Xpert Ultra and culture results, and radiographic abnormalities were binary or categorical variables. Participants were classified as having bacteriologically confirmed TB if they had a positive Xpert Ultra test and/or mycobacterial culture. Participants were classified as having empirically diagnosed TB if they had symptoms and chest radiograph consistent with TB, without bacteriologic confirmation.

We calculated sensitivity, specificity, positive and negative predictive values (PPV, NPV), and positive and negative likelihood ratios of varying CRP thresholds (≥1.0, ≥2.0, ≥5.0, ≥10.0 mg/L) in the diagnosis of TB in the total cohort of symptomatic patients, and stratified by symptoms (cough, fever, and/or night sweats vs weight loss only). We also calculated these test characteristics in the diagnosis of bacteriologically confirmed TB. In addition, we conducted a log-transformation of the raw CRP values to stabilize the data variance, and then assessed predictors of log (CRP) in the total cohort using generalized linear models. We further compared baseline demographic and clinical characteristics among patients diagnosed with TB, stratified by high (≥3 mg/L) versus low (<3 mg/L) CRP using the chi-squared test for categorical variables and *t*-test or Wilcoxon rank-sum test for continuous variables. Finally, we analyzed the number and proportion of patients who would have been hypothetically eligible for same-day ART, based on symptoms (cough, fever, and/or night sweats vs isolated weight loss) and varying CRP thresholds. For each hypothetical scenario, we determined the proportion who would have been eligible for same-day ART (with and without undiagnosed TB).

## RESULTS

Five hundred participants were enrolled in the trial from 6 November 2017 to 16 January 2020; of these, 498 (99.6%) had a CRP test result at HIV diagnosis and were included in the analysis. Median age was 37 years (IQR: 30, 45), 234 (46.8%) were female, and median BMI was 20.7 (IQR 18.7, 22.9). Median hemoglobin was 11.2 g/dL (IQR: 9.9, 12.3) in females and 12.7 g/dL (10.6, 14.3) in males; median CD4 count was 276 cells/mm^3^ (IQR: 128, 426) and median CRP was 3.9 mg/L (IQR: 1.0, 18.7) (see Table 1). A total of 260 participants (52.2%) reported cough, fever, and/or night sweats (± weight loss) and 238 (47.8%) reported isolated weight loss.

**Table 1.** Baseline Characteristics by TB Status

Variable	Total Cohort	TB Cohort	Non-TB Cohort	*P V*alue
	(n = 498)	(n = 87)	(n = 411)	
Female—no. (%)	234 (47.0)	33 (37.9)	201 (48.9)	.081
Age (y)—median (IQR)	37 (30, 45)	36 (31, 43)	37 (30, 46)	.531
Cough, fever, and/or night sweats—no. (%)	260 (52.2)	78 (89.7)	182 (44.3)	<.001
Body mass index—median (IQR)	20.7 (18.7, 22.9)	18.9 (17.7, 20.6)	21.1 (19.2, 23.3)	<.001
CD4 count category—no. (%)	…	…	…	.028
<100 cells/mm^3^	100 (20.3)	25 (29.4)	75 (18.4)	…
100–349 cells/mm^3^	224 (45.4)	39 (45.9)	185 (45.3)	…
350–499 cells/mm^3^	84 (17.0)	14 (16.5)	70 (17.2)	…
≥500 cells/mm^3^	85 (17.2)	7 (8.2)	78 (19.1)	…
CD4 count—median (IQR)	276 (128, 426)	170 (86, 342)	288 (140, 444)	<.001
Hemoglobin (g/dL)—median (IQR)	11.9 (10.1, 13.4)	9.4 (8.3, 11.0)	12.2 (10.7, 13.7)	<.001
C-reactive protein (mg/L)—no. (%)	…	…	…	…
≥1.0	371 (74.5)	83 (95.4)	288 (70.1)	<.001
≥2.0	308 (61.8)	76 (87.4)	232 (56.4)	<.001
≥3.0	267 (53.6)	75 (86.2)	192 (46.7)	<.001
≥5.0	226 (45.4)	66 (75.9)	160 (38.9)	<.001
≥10.0	175 (35.1)	62 (71.3)	113 (27.5)	<.001
C-reactive protein (mg/L)—median (IQR)	3.9 (1.0, 18.7)	33.0 (5.1, 85.5)	2.6 (0.8, 11.7)	<.001

Abbreviation: IQR, interquartile range.

At enrollment, 87 (17.5%) participants were diagnosed with TB; 67 (13.5%) had bacteriologically confirmed TB and 20 (4.0%) had empirically diagnosed TB. Those diagnosed with baseline TB were more likely to report cough, fever, and/or night sweats (vs isolated weight loss), and had lower BMI, hemoglobin, and CD4 cell counts compared to those not diagnosed with TB (see Table 1). Median CRP was 33.0 mg/L (IQR: 5.1, 85.5) in those diagnosed with TB, and 2.6 mg/L (IQR: 0.8, 11.7) in those without TB. Any radiographic abnormality consistent with pulmonary TB was detected in 45 (51.7%) of participants diagnosed with TB and 27 (6.6%) of those without TB.

In the multivariable analysis, higher CRP was associated with report of cough, fever, and/or night sweats (vs isolated weight loss) and being diagnosed with TB. A lower CRP was associated with higher CD4 cell count, higher hemoglobin levels, and female sex (see Table 2).

**Table 2.** Predictors of Elevated C-Reactive Protein

Variable	Univariable Analysis		Multivariable Analysis	
	Coefficients (95% CI)	*P* Value	Adjusted Coefficients (95% CI)	*P* Value
Female	−0.20 (−0.53, 0.14)	0.258	−0.46 (−0.80, −0.12)	.008
Age (per decade)	0.08 (−0.09, 0.25)	0.344	0.06 (−0.09, 0.22)	.412
Cough, fever, and/or night sweats (vs isolated weight loss)	1.24 (0.92, 1.56)	<0.001	0.80 (0.47, 1.12)	<.001
Body mass index (per unit)	−0.07 (−0.12, −0.03)	0.001	0.03 (−0.01, 0.08)	.176
CD4 count (per 50 cells/mm^3^)	−0.11 (−0.15, −0.08)	<0.001	−0.08 (−0.11, −0.05)	<.001
Hemoglobin (per g/dL)	−0.03 (−0.04, −0.02)	<0.001	−0.02 (−0.03, −0.01)	<.001
Diagnosed with tuberculosis	1.60 (1.13, 2.08)	<0.001	0.69 (0.21, 1.17)	.005

Among participants with TB, we compared baseline demographic and clinical characteristics between those with lower CRP (<3 mg/L) versus higher CRP (≥3 mg/L). There was no significant difference in any of the variables assessed, including sex, age, symptoms, BMI, CD4 count, hemoglobin, sputum results, or radiographic findings (see Table 3).

**Table 3.** Baseline Demographic and Clinical Characteristics Among Participants With Tuberculosis, by C-Reactive Protein Level

Variable	All Participants With TB (n = 87)	CRP ≥3 (n = 75)	CRP <3 (n = 12)	*P V*alue
Female—no. (%)	33 (37.9)	28 (37.3)	5 (41.7)	.76
Age at enrollment (y)—median (IQR)	36 (31, 43)	36 (31, 42)	39 (33, 45)	.662
Isolated weight loss (no cough, fever, or night sweats)—no (%)	9 (10.3)	7 (9.3)	2 (16.7)	.605
Body mass index—median (IQR)	18.9 (17.7, 20.6)	18.8 (17.7, 20.7)	19.4 (17.7, 20.1)	.849
CD4 count—median (IQR)	170 (86, 342)	177 (88, 317)	122.5 (70.8, 382.3)	.686
CD4 count category—no. (%)	…	…	…	.246
<100 cells/mm^3^	25 (29.4)	20 (27.4)	5 (41.7)	…
100–349 cells/mm^3^	39 (45.9)	36 (49.3)	3 (25)	…
≥350 cells/mm^3^	21 (24.7)	17 (23.2)	4 (33.4)	…
Hemoglobin (g/dL)—median (IQR)	9.4 (8.3, 11.0)	9.4 (8.2, 10.9)	9.1 (8.8., 11.7)	.483
Xpert Ultra positive (2 samples)	37 (42.5)	33 (44.0)	4 (33.3)	.704
Xpert Ultra positive (1 sample)	55 (63.2)	48 (64.0)	7 (58.3)	.753
Xpert Ultra and/or culture positive	67 (77.0)	57 (76.0)	10 (83.3)	.725
Any radiographic abnormality consistent with TB	45 (51.7)	40 (53.3)	5 (41.7)	.66

Abbreviation: IQR, interquartile range.

We assessed the impact of a range of CRP thresholds on the PPV and NPV of baseline TB in this cohort of symptomatic patients (see Table 4). As the CRP threshold increased from ≥1 mg/L, to ≥3 mg/L, ≥ 5 mg/L and ≥10 mg/L, the PPV for TB in the total cohort increased from 22.4% to 28.1%, 29.2%, and 35.4%, respectively, and the NPV decreased from 96.9% to 94.8%, 92.3%, and 92.3%. Supplementary Table 1 includes these results in participants with bacteriologically confirmed TB.

**Table 4.** Utility of C-Reactive Protein Thresholds for Ruling In or Ruling Out Tuberculosis

CRP Threshold	Proportion of Study Population (%)	Sensitivity (95% CI)	Specificity (95% CI)	PPV (95% CI)	NPV (95% CI)	Positive LR (95% CI)	Negative LR (95% CI)
Total cohort (participants with cough, fever, night sweats, and/or weight loss)							
≥1.0	74.5	95.4 (88.6–98.7)	29.9 (25.5–34.6)	22.4 (18.2–27.0)	96.9 (92.1–99.1)	1.4 (1.3–1.5)	0.2 (0.1–0.4)
≥2.0	61.8	87.4 (78.5–93.5)	43.6 (38.7–48.5)	24.7 (20–29.9)	94.2 (89.9–97.1)	1.5 (1.4–1.7)	0.3 (0.2–0.5)
≥3.0	53.6	86.2 (77.1–92.7)	53.3 (48.3–58.2)	28.1 (22.8–33.9)	94.8 (91.1–97.3)	1.8 (1.6–2.1)	0.3 (0.2–0.4)
≥5.0	45.4	75.9 (65.5–84.4)	61.1 (56.2–65.8)	29.2 (23.4–35.6)	92.3 (88.4–95.2)	1.9 (1.6–2.3)	0.4 (0.3–0.6)
≥10.0	35.1	71.3 (60.6–80.5)	72.5 (67.9–76.8)	35.4 (28.4–43.0)	92.3 (88.8–94.9)	2.6 (2.1–3.2)	0.4 (0.3–0.6)
Participants with cough, fever, and/or nights sweats ± weight loss (excludes participants with isolated weight loss)							
≥1 .0	81.9	94.9 (87.4–98.6)	23.6 (17.7–30.5)	34.7 (28.4–41.5)	91.5 (79.6–97.6)	1.2 (1.1–1.4)	0.2 (0.1–0.6)
≥2.0	71.9	87.2 (77.7–93.7)	34.6 (27.7–42)	36.4 (29.5–43.7)	86.3 (76.2–93.2)	1.3 (1.2–1.5)	0.4 (0.2–0.7)
≥3.0	65.4	87.2 (77.7–93.7)	44.0 (36.6–51.5)	40.0 (32.6–47.8)	88.9 (80.5–94.5)	1.6 (1.3–1.8)	0.3 (0.2–0.5)
≥5.0	58.8	80.8 (70.3–88.8)	50.5 (43.1–58)	41.2 (33.3–49.4)	86.0 (77.9–91.9)	1.6 (1.4–2.0)	0.4 (0.2–0.6)
≥10.0	49.6	75.6 (64.6–84.7)	61.5 (54.1–68.6)	45.7 (36.9–54.7)	85.5 (78.3–91.0)	2.0 (1.6–2.5)	0.4 (0.3–0.6)
Patients with isolated weight loss (excludes participants with cough, fever, or night sweats)							
≥1.0	66.4	100 (66.4–100)	34.9 (28.8–41.5)	5.7 (2.6–10.5)	100.0 (95.5–100.0)	1.5 (1.4–1.7)	0
≥2.0	50.8	88.9 (51.8–99.7)	50.7 (44.0–57.3)	6.6 (2.9–12.6)	99.1 (95.3–100.0)	1.8 (1.4–2.3)	0.2 (0.0–1.4)
≥3.0	40.8	77.8 (40.0–97.2)	60.7 (54.0–67.1)	7.2 (3.0–14.3)	98.6 (95.0–99.8)	2 (1.3–2.9)	0.4 (0.1–1.2)
≥5.0	30.7	33.3 (7.5–70.1)	69.4 (63.0–75.3)	4.1 (0.9–11.5)	96.4 (92.3–98.7)	1.1 (0.4–2.8)	1 (0.6–1.5)
≥10.0	19.3	33.3 (7.5–70.1)	81.2 (75.6–86.1)	6.5 (1.4–17.9)	96.9 (93.3–98.8)	1.8 (0.7–4.6)	0.8 (0.5–1.3)

Abbreviations: CI, confidence interval; CRP, C-reactive protein; LR, likelihood ration; NPV, negative predictive value; PPV, positive predictive value.

Among the 260 participants with cough, fever, and/or night sweats (± weight loss), the prevalence of baseline TB was 30%. The PPV for TB increased from 34.7% to 40.0%, 41.2%, and 45.7% as the CRP threshold increased from ≥1 mg/L, to ≥3 mg/L, ≥ 5 mg/L, and ≥10 mg/L, respectively, and the NPV decreased from 91.5% to 88.9%, 86.0%, and 85.5%.

Among the 238 participants with isolated weight loss (ie, excluding those with cough, fever, and/or night sweats), the prevalence of TB was 3.8%. The PPV was only 6.5% at CRP threshold of ≥10 mg/L. The NPV for TB decreased from 100% at a CRP threshold of ≥1 mg/L, to 98.6%, 96.4%, and 96.9% at thresholds of ≥3 mg/L, ≥ 5 mg/L, and ≥10 mg/L, respectively.

**Figure 1.**
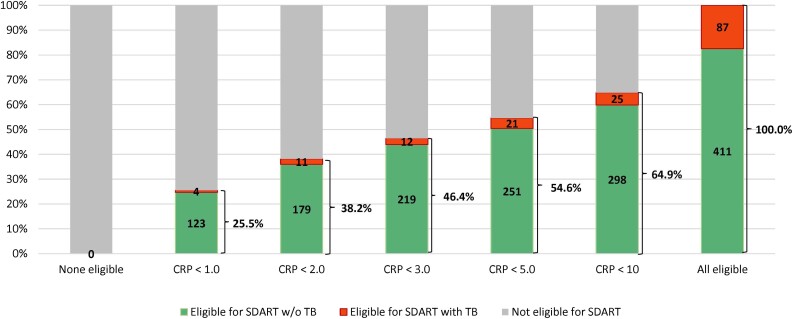
Impact of CRP-based ART eligibility thresholds on same-day ART qualification and undiagnosed tuberculosis (n = 498).

CRP testing was conducted retrospectively, so results were not available at enrollment in the parent trial; in accordance with the study protocol, all 498 participants had TB testing before ART initiation. If we had implemented the WHO-recommended strategy of ART initiation before completion of TB testing, then 87 (17.5%) participants would have started ART with untreated TB. If we had included CRP concentrations in our same-day ART eligibility algorithm, then with CRP thresholds of <1, <2, <3, <5, and <10 mg/L, a total of 127 (25.5%), 190 (38.2%), 231 (46.4%), 272 (54.6%), and 323 (64.9%) participants would have been eligible for same-day ART (see Figure 1). A total of 4 (0.8%), 11 (2.2%), 12 (2.4%), 21 (4.2%), and 25 (5.0%) participants, respectively, would have had untreated active TB at ART initiation (active TB in spite of having CRP levels below the cutoff).

**Figure 2.**
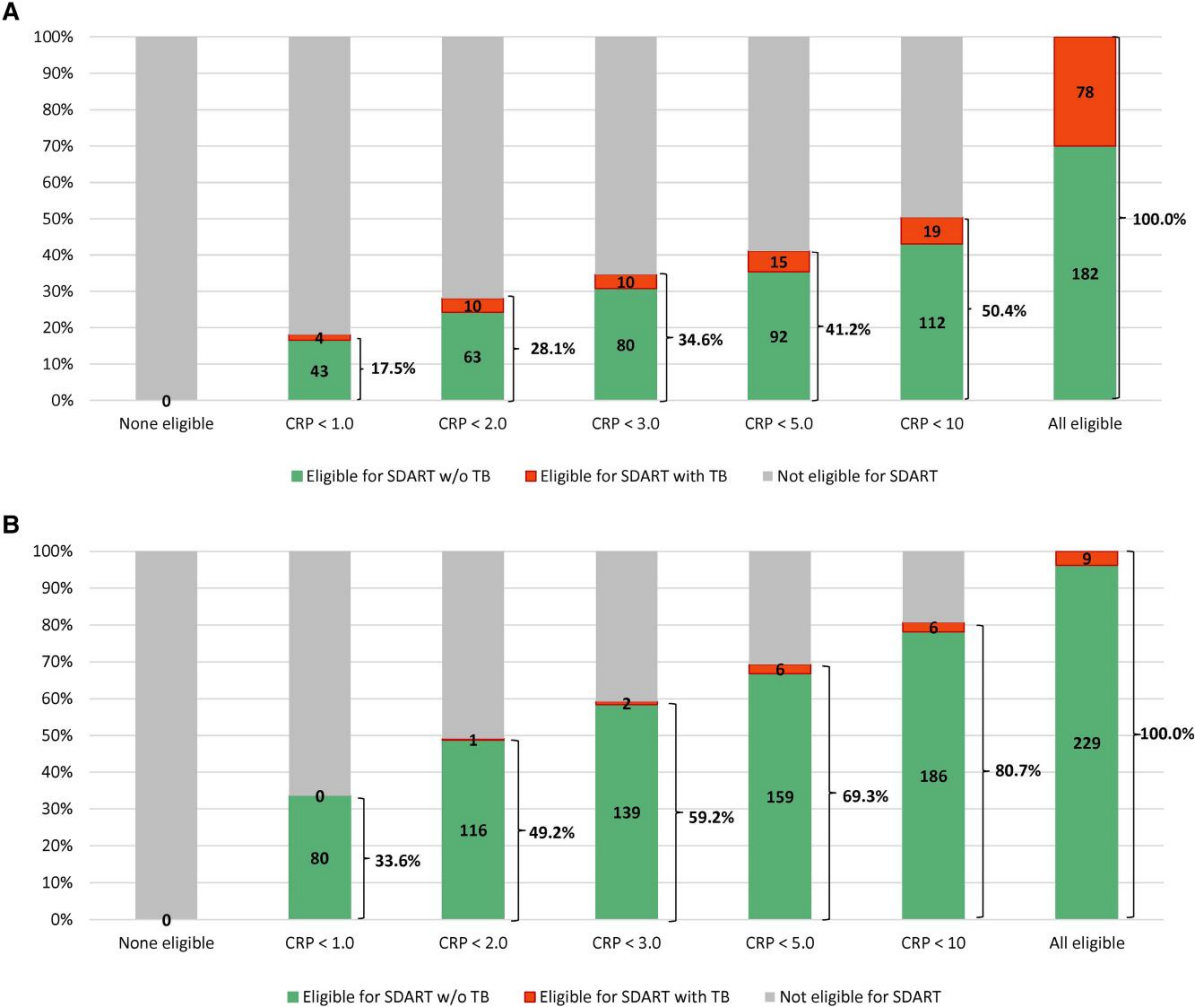
*A*, Impact of CRP-based eligibility criteria on same-day ART eligibility and untreated TB in participants with cough, fever, and/or night sweats at HIV diagnosis (n = 260), *B*, Impact of CRP-based eligibility criteria on same-day ART eligibility and untreated TB in participants with isolated weight loss at HIV diagnosis (n = 238).

Among the 260 participants who reported cough, fever, and/or night sweats (± weight loss), if same-day ART had been initiated while TB testing was under way, then 78 (30.0%) would have started ART with untreated TB. If CRP thresholds of <1, <2, <3, <5, and <10 mg/L had been used as eligibility thresholds, then 47 (17.5%), 73 (28.1%), 90 (34.6%), 107 (41.2%), and 131 (50.4%) patients, respectively, would have been eligible for same-day ART (see Figure 2*A*). A total of 4 (1.5%), 10 (3.8%), 10 (3.8%), 15 (5.8%), and 19 (7.3%) patients, respectively, would have had untreated TB at ART initiation.

Among the 238 participants who reported isolated weight loss, 9 (3.8%) were diagnosed with baseline TB. If CRP thresholds of <1, <2, <3, <5, and <10 mg/L had been used as same-day ART eligibility thresholds, then 80 (33.6%), 117 (49.2%), 141 (59.2%), 165 (69.3%), and 192 (80.7%) patients, respectively would have been eligible for same-day ART (see Figure 2*B*). A total of 0 (0.0%), 1 (0.4%), 2 (0.8%), 6 (2.5%), and 6 (2.5%) patients, respectively, would have had untreated TB at ART initiation.

## DISCUSSION

We found that CRP concentrations can be used to stratify symptomatic patients into high or low risk of TB at HIV diagnosis, facilitating decision making about same-day ART initiation. The WHO currently recommends the W4SS screen at HIV diagnosis, with 2 guideline-approved algorithms for symptomatic patients—either same-day ART while awaiting TB test results or deferral of ART until completion of TB testing [1]. However, these approaches either result in ART initiation in patients with undiagnosed TB or unnecessary delays in ART for those without TB. In our cohort, the prevalence of baseline TB was 17.5%, and the PPV of CRP was 22.4%, 28.1%, 29.2%, and 35.4% at thresholds ≥1 mg/L, ≥3 mg/L, ≥5 mg/L, and ≥10 mg/L, respectively. With the use of CRP-based eligibility criteria, one-quarter to two-thirds of symptomatic patients could be eligible for same-day ART, depending on the CRP threshold used, with a 3- to 20-fold reduction in the proportion of patients with undiagnosed TB at ART initiation, compared with the WHO strategy of same-day ART before completion of TB testing.

We also found that baseline TB prevalence varied by W4SS symptom category. About half of the total cohort reported cough, fever, and/or nights sweats (± weight loss), and nearly half reported isolated weight loss. The prevalence of TB in those with cough, fever, and/or night sweats was almost 10-fold higher than that of the participants who reported isolated weight loss (30.0% vs 3.8%). Among participants with cough, fever, and/or night sweats, the PPV of CRP was 40.0%, 41.2%, and 45.7% at thresholds ≥3 mg/L, ≥5 mg/L and ≥10 mg/L, respectively. If CRP values under these thresholds were used to define same-day ART eligibility, then one-third to one-half of this subgroup would be eligible for same-day ART, with a 4- to 8-fold reduction in the proportion of patients with undiagnosed TB at ART initiation, compared with the strategy of same-day ART testing before completion of TB testing.

In contrast, among participants with isolated weight loss, the PPV is low at all CRP thresholds, but the NPV is high; with CRP thresholds of <1, <2, or <3, up to about 60% of patients would be eligible for same-day ART initiation, with <1% of patients having baseline TB and CRP concentrations below these cutoffs. A study conducted among ART-naïve patients in South Africa also reported that the NPV of CRP <1 mg/L was 100% [21]. These data suggest that clinicians can confidently commence ART in patients with a new diagnosis of HIV with isolated weight loss as a symptom and low CRP. In fact, from our study, less than 1% of patients with this clinical picture and a CRP <3 mg/L were found to have TB.

Multiple studies of ART-naïve patients in African cohorts that included mycobacterial culture have reported a TB prevalence similar to ours [22–26]. Most studies that reported 4WSS results combined all 4 symptoms [27]. Among those that reported individual symptoms, other studies have also reported a lower TB prevalence among patients reporting isolated weight loss, compared with cough, fever, and/or night sweats. In a clinical trial cohort of newly diagnosed patients from South Africa and Kenya, the PPV value of cough and fever was >30% versus about 5% for isolated weight loss [28]. A cohort study from Ethiopia found that cough was the most predictive of active TB [26].

These data indicate that if the WHO recommendation to initiate same-day ART in symptomatic patients while TB testing under way is implemented, a substantial proportion of patients with active TB will initiate ART prior to TB treatment [1]. However, data on the safety of this approach are limited [13]. A clinical trial is currently under way in patients who report at least 1 symptom on the W4SS at HIV diagnosis in Lesotho and Malawi to compare outcomes with a strategy of waiting for TB test results versus immediately initiating ART [29]. However, to date, same-day ART studies have included only a small number of participants with TB and nearly all received efavirenz-based regimens [6, 8, 10, 12, 13]. Currently, most persons with HIV in high-burden settings receive dolutegravir-based regimens, which result in faster improvements in immune function. ART-mediated immune restoration has been associated with pulmonary dysfunction, even in patients who initiate timely treatment without clinical evidence of immune reconstitution inflammatory syndrome [30, 31]. Initiating dolutegravir-based ART in the presence of untreated TB could potentially provoke further declines in pulmonary function. This is a nontrivial concern because there is a growing body of literature documenting substantial morbidity and mortality associated with post-TB lung disease [32, 33].

We are not aware of any HIV program that uses CRP in decision making about the timing of ART initiation. Multiple studies have demonstrated CRP has similar sensitivity but greater specificity than the W4SS, but these studies were conducted to assess alternative strategies for more efficient use of TB diagnostic testing, or the potential for CRP-based screening strategies to improve the update of TB preventive treatment [17–21]. Current WHO guidelines provide a conditional recommendation (low-certainty evidence for test accuracy) for CRP with a cutoff of >5 mg/L to screen for TB disease in people with HIV [1]. However, CRP is not included in the algorithms for same-day ART initiation.

The sensitivity and specificity of the W4SS are about 82% and 42%, respectively [20, 27]. In a systematic review and individual participant data meta-analysis commissioned by the WHO, the investigators found that among outpatients not on ART, the sensitivities of CRP (≥10 mg/L) and a sequential strategy of W4SS followed by CRP (≥5 mg/L) were similar to W4SS alone, but the specificities were higher [20]. A study from Uganda found that point-of-care CRP had 89% sensitivity and 72% specificity for culture-confirmed TB among patients with CD4 count ≤350 cells/mm^3^ (regardless of symptoms) who were initiating ART [18]. A study from South Africa reported that the sensitivity and specificity of CRP >5 for culture-positive TB were 91% and 59%, respectively, among outpatients systematically tested for TB, regardless of symptoms [17]. Among our cohort of patients with positive W4SS symptom screens, the sensitivity of CRP was higher at threshold of ≥3 mg/L versus ≥5 mg/L (86% vs 76%), with a slight decline in specificity (53% vs 61%) [20].

These findings highlight the potential utility of CRP testing to inform decision making about same-day ART initiation in patients who present with TB symptoms at HIV diagnosis. CRP is available as a simple, rapid, low-cost (<$US 2), point-of-care test, which is available from a variety of manufacturers [16]. Further study is necessary to define optimal CRP thresholds, which may potentially vary by number and type of symptoms. Moreover, CRP would not replace diagnostic testing for TB or other opportunistic infections and noncommunicable diseases.

Our study was conducted among nonpregnant participants in a large, urban clinic, which may limit the generalizability of our findings. Additionally, we completed CRP testing retrospectively on stored samples, whereas using CRP to guide clinical decisions about same-day ART would necessitate use of a point-of-care assay. It is also important to note that we used a more aggressive diagnostic approach for TB than is provided in many high-burden settings. However, in settings without access to mycobacterial culture and chest radiographs, the detection of a high CRP level (>5 mg/L or >10 mg/L, for example) can alert providers to a potential case of TB or other opportunistic infection, even if sputum smear or Xpert MTB/RIF testing is negative. Among patients with isolated weight loss without respiratory symptoms or fever, the clinician and program can have even greater confidence in commencing ART (and not missing a TB diagnosis) in the presence of a low CRP.

## CONCLUSIONS

In conclusion, our results demonstrate that CRP testing can be useful in stratifying patients with non-meningitic symptoms into high or low risk of TB at HIV diagnosis, facilitating decision making about same-day ART initiation.

## Supplementary Material

ofae356_Supplementary_Data

## Supplementary Data

Supplementary materials are available at *Open Forum Infectious Diseases* online. Consisting of data provided by the authors to benefit the reader, the posted materials are not copyedited and are the sole responsibility of the authors, so questions or comments should be addressed to the corresponding author.

## Notes

***Acknowledgments.*** We thank the patients who participated in this study and the GHESKIO staff who cared for them. We also thank the members of the Data Safety Monitoring Board, the Community Advisory Board, and the ethics committees for their expertise and advice.

***Author contributions.*** All authors were involved in drafting the article, and all approved the final version to be published. Study conception and design: K.D., V.R.R., N.D., C.R., P.S., S.P., K.F.W., P.J., J.W.P., and S.P.K. Acquisition of data: N.D., A.A., E.D., C.R., P.S., K.L., S.P., and V.R.. Analysis and interpretation of data: K.D., V.R.R., N.D., H.A., Y.G., J.L., M.D.M., K.F.W., A.L.B., P.Y.C., J.W.P., and S.P.K.

***Financial support.*** This study was funded by a grant from the National Institute of Allergy and Infectious Diseases (R01AI131998; primary investigator: S.K.). K.D. was supported by NIAID (K23AI131913) and a Doris Duke Charitable Foundation Clinical Scientist Development Award.
