# Supplementary material for: Potential Utility of C-reactive Protein for Tuberculosis Risk Stratification Among Patients With Non-Meningitic Symptoms at HIV Diagnosis in Low- and Middle-income Countries
Source: Open Forum Infect Dis. 2024 Jul 3;11(7):ofae356. doi: 10.1093/ofid/ofae356 (PMC11252845; doi:10.1093/ofid/ofae356)
Supplement: ofae356_Supplementary_Data [file ofae356_supplementary_data.docx]

**Supplemental Table 1. Utility of C-Reactive Protein Thresholds for Ruling In or Ruling Out Bacteriologically-Confirmed Tuberculosis***

| **CRP Threshold** | **Proportion of Total Study Population (%)** | **Sensitivity (95% CI)** | **Specificity (95% CI)** | **PPV**  **(95% CI)** | **NPV**  **(95% CI)** | **Positive LR (95% CI)** | **Negative LR (95% CI)** |
| --- | --- | --- | --- | --- | --- | --- | --- |
| ***Patients with Cough, Fever, Night Sweats, and/or Weight Loss*** | | | | | | | |
| ≥1.0 | 74.5 | 94.0 (85.4-98.3) | 28.5 (24.3-33.1) | 17.0 (13.3-21.2) | 96.9 (92.1-99.1) | 1.3 (1.2-1.4) | 0.2 (0.1-0.5) |
| ≥2.0 | 61.8 | 86.6 (76-93.7) | 42.0 (37.3-46.8) | 18.8 (14.6-23.7) | 95.3 (91.2-97.8) | 1.5 (1.3-1.7) | 0.3 (0.2-0.6) |
| ≥3.0 | 53.6 | 85.1 (74.3-92.6) | 51.3 (46.4-56.1) | 21.3 (16.6-26.8) | 95.7 (92.2-97.9) | 1.7 (1.5-2) | 0.3 (0.2-0.5) |
| ≥5.0 | 45.4 | 74.6 (62.5-84.5) | 59.2 (54.4-63.8) | 22.1 (16.9-28.1) | 93.8 (90.2-96.3) | 1.8 (1.5-2.2) | 0.4 (0.3-0.7) |
| ≥10.0 | 35.1 | 70.1 (57.7-80.7) | 70.3 (65.7-74.6) | 26.9 (20.4-34.1) | 93.8 (90.6-96.2) | 2.4 (1.9-2.9) | 0.4 (0.3-0.6) |
| ***Patients with Cough, Fever, and/or Nights Sweats +/- Weight Loss (Excludes Patients with Only Weight Loss)*** | | | | | | | |
| ≥1.0 | 81.9 | 93.2 (83.5-98.1) | 21.4 (15.9-27.7) | 25.8 (20.1-32.2) | 91.5 (79.6-97.6) | 1.2 (1.1-1.3) | 0.3 (0.1-0.8) |
| ≥2.0 | 71.9 | 86.4 (75.0-94.0) | 32.3 (25.9-39.3) | 27.3 (21.0-34.3) | 89 (79.5-95.1) | 1.3 (1.1-1.5) | 0.4 (0.2-0.8) |
| ≥3.0 | 65.4 | 86.4 (75.0-94.0) | 40.8 (33.9-47.9) | 30.0 (23.2-37.5) | 91.1 (83.2-96.1) | 1.5 (1.3-1.7) | 0.3 (0.2-0.6) |
| ≥5.0 | 58.8 | 79.7 (67.2-89.0) | 47.3 (40.2-54.4) | 30.7 (23.5-38.7) | 88.8(81.2-94.1) | 1.5 (1.3-1.8) | 0.4 (0.3-0.7) |
| ≥10.0 | 49.6 | 74.6 (61.6-85.0) | 57.7 (50.6-64.6) | 34.1 (26-43) | 88.5 (81.8-93.4) | 1.8 (1.4-2.2) | 0.4 (0.3-0.7) |
| ***Patients with Weight Loss Only (Excludes Patients with Cough, Fever, or Night Sweats)*** | | | | | | | |
| ≥1.0 | 66.4 | 100 (63.1-100) | 34.8 (28.6-41.3) | 5.1 (2.2-9.7) | 100.0 (95.5-100.0) | 1.5 (1.4-1.7) | 0.0 (0.0- -) |
| ≥2.0 | 50.8 | 87.5 (47.3-99.7) | 50.4 (43.8-57.1) | 5.8 (2.4-11.6) | 99.1 (95.3-100.0) | 1.8 (1.3-2.4) | 0.2 (0-1.6) |
| ≥3.0 | 40.8 | 75.0 (34.9-96.8) | 60.4 (53.8-66.8) | 6.2 (2.3-13) | 98.6 (95-99.8) | 1.9 (1.2-2.9) | 0.4 (0.1-1.4) |
| ≥5.0 | 30.7 | 37.5 (8.5-75.5) | 69.6 (63.2-75.4) | 4.1 (0.9-11.5) | 97.0 (93.1-99.0) | 1.2 (0.5-3.1) | 0.9 (0.5-1.5) |
| ≥10.0 | 19.3 | 37.5 (8.5-75.5) | 81.3 (75.7-86.1) | 6.5 (1.4-17.9) | 97.4 (94-99.1) | 2.0 (0.8-5.1) | 0.8 (0.4-1.3) |

*Bacteriologically-confirmed tuberculosis required a positive Xpert Ultra test and/or mycobacterial culture
